# Using an Ionic Liquid to Reduce the Electrical Percolation Threshold in Biobased Thermoplastic Polyurethane/Graphene Nanocomposites

**DOI:** 10.3390/polym11030435

**Published:** 2019-03-06

**Authors:** Nora Aranburu, Itziar Otaegi, Gonzalo Guerrica-Echevarria

**Affiliations:** POLYMAT and Polymer Science and Technology Department, Faculty of Chemistry, University of the Basque Country UPV/EHU, Paseo Manuel de Lardizabal 3, 20018 Donostia, Spain; nora.aramburu@ehu.eus (N.A.); itziar.otaegi@ehu.eus (I.O.)

**Keywords:** nanocomposites, graphene, melt processing, mechanical properties, electrical conductivity

## Abstract

Biobased thermoplastic polyurethane (bTPU)/unmodified graphene (GR) nanocomposites (NCs) were obtained by melt-mixing in a lab-scaled conventional twin-screw extruder. Alternatively, GR was also modified with an ionic liquid (GR-IL) using a simple preparation method with the aim of improving the dispersion level. XRD diffractograms indicated a minor presence of well-ordered structures in both bTPU/GR and bTPU/GR-IL NCs, which also showed, as observed by TEM, nonuniform dispersion. Electrical conductivity measurements pointed to an improved dispersion level when GR was modified with the IL, because the bTPU/GR-IL NCs showed a significantly lower electrical percolation threshold (1.99 wt%) than the bTPU/GR NCs (3.21 wt%), as well as higher conductivity values. Young’s modulus increased upon the addition of the GR (by 65% with 4 wt%), as did the yield strength, while the ductile nature of the bTPU matrix maintained in all the compositions, with elongation at break values above 200%. This positive effect on the mechanical properties caused by the addition of GR maintained or slightly increased when GR-IL was used, pointing to the success of this method of modifying the nanofiller to obtain bTPU/GR NCs.

## 1. Introduction

Polymer nanocomposites (NCs) containing carbon-based electrically conductive nanoparticles have gained a certain advantage over other hybrid polymer systems because, in addition to the other usual improvements in mechanical and transport properties, they offer enhanced electrical and thermal conductivity. As a result, these NCs are particularly suitable for different applications such as low-cost, light-weight, EMI-shielded computer housing and cables, antistatic packaging, high-strength automotive and aerospace components, high-barrier packaging, and smart clothing/personal sensor systems [1].

Graphene (GR) is a two-dimensional carbon nanofiller with a one-atom-thick planar sheet of sp^2^ bonded carbon atoms densely packed in a honeycomb crystal lattice [2]. The good thermal conductivity and excellent mechanical and electronic transport properties of graphene make it an ideal candidate for producing thermally and electrically-conductive reinforced NCs [3,4]. However, in order to achieve these goals, the graphene must be efficiently dispersed in the polymer matrix, a process which is hindered by the strong intrinsic van der Waals attraction and π–π stacking which tends to cause the reaggregation of the graphene sheets [5].

Among the different covalent and noncovalent modifications of graphene which are used in order to overcome the aforementioned restacking problem [6], the use of ionic liquids (IL) as dispersing agents has attracted the attention of the scientific community as their use is considered simple, ecofriendly, and highly efficient [7,8]. ILs are believed to interact with graphene by cation–π stacking and π–π interactions which prevent the reaggregation of graphene sheets while preserving the electronic structure of the graphene and its intrinsic properties [9,10]. As a result, ILs have been successfully used as dispersing agents for graphene in several thermoplastic matrices, including poly(vinyl alcohol) [11], a copolyamide [12], polylactide [9], polystyrene [13], poly(methyl methacrylate) [14], and polyvinylidene fluoride [7].

In the search for potential polymer matrices, thermoplastic polyurethanes (TPUs) have attracted much attention in the recent years [15,16]. This is because they are linear block copolymers with hard and soft segments and, thus, depending on the composition of the reactants used, such as isocyanates, polyols, and chain extenders, they can offer a variety of properties, rendering them suitable for a broad range of applications including automotive, electronics, sports goods, footwear, and medical devices. The addition of graphene-like structures to TPUs would further broaden the application potential of these polymers as it would make it possible to obtain electrically conductive TPUs with improved mechanical and other properties. This is why several papers on TPU-based NCs containing graphene [17,18,19,20,21,22,23,24,25,26,27,28,29,30,31,32] have been included in the bibliography. However, most of these studies focus on systems prepared by in situ polymerization [21,22,26] and solvent mixing [18,19,20,23,26,30,31,32]. Unfortunately, only a few studies are devoted to TPU/graphene NCs prepared by melt blending [24,25,26,27,28,29,31], which is the most practical, versatile, economical, and environmentally-friendly method as it can be easily adapted to existing conventional plastic processing equipment [33] and does not require the use of solvents or monomers [34]. Furthermore, in all these last cases, nonindustrial recirculating mixing equipment was used during processing. Even though the reinforcement effect of the graphene has been detailed [24,25,26,27,28,29,31] in several studies, only a few characterize the electrical conductivity of the NCs [26,28,31] in their analysis of the contribution of graphene as a conductive nanofiller. Moreover, the use of IL as a dispersing agent to improve the dispersion of graphene in TPU-based NCs has not been studied to the best of our knowledge.

Finally, the development and production of polymeric materials from renewable resources have become subjects of considerable interest in the academic and industrial sectors in recent years, due to their low cost and a growing concern about global warming, carbon emissions and the nonavailability of certain petrochemical raw materials [35,36]. In fact, biobased TPUs synthesized with polyols obtained from natural renewable resources are already commercially available [37,38]. The behavior of these commercial biobased TPUs as potential matrices for the preparation of NCs containing graphene has not, to our knowledge, been studied.

In this work, electrically conductive NCs with improved mechanical properties were obtained using conventional industrial-like melt-mixing equipment with a biobased TPU (bTPU) as the polymeric matrix. Neat GR and GR modified with an IL (GR-IL)—1-butyl-3-methylimidazolium tetrafluoroborate (BMITFB)—were used as nanofillers. The nanostructure, electrical conductivity, and thermal and mechanical properties of the bTPU/GR and bTPU/GR-IL NCs were studied and compared.

## 2. Materials and Methods

The polymer used in this work was a linear, biobased aromatic polyurethane, Pearlbond^®^ Eco D590, based on specialty polyols from renewable sources and kindly supplied by Merquinsa (Barcelona, Spain). Its chemical structure, analyzed by NMR, consists of methylene diphenyl 4,4’-diisocyanate (MDI) (4 wt%) and polybutylene sebacate (PBSe) (96 wt%). Expanded graphene (GR), comprising 1–2 layers, with a purity > 98.5% and density < 0.2 g/cm^2^, was purchased from Avanzare (Navarrete, Spain). The ionic liquid (IL) was 1-butyl-3-methylimidazolium tetrafluoroborate (purity ≥ 98%) from Aldrich (Saint Louis, MO, USA).

The modification of GR with IL was performed as follows: first, GR was sonicated in ethanol for 15 min after which the IL was added (in 1:1 ratio). Next, the mixture was sonicated for 1 h. Then it was dried in an oven at 60 °C for 24 h in order to remove the ethanol and obtain the GR-IL powder.

The composition of the prepared bTPU NCs, as well as their real GR content are listed on Table 1. The NCs were first melt-mixed by extrusion and then injection- or compression-molded to obtain standard test specimens.

The extruder used was a Collin ZK25 corotating twin screw extruder-kneader (Collin, Maitenbeth, Germany) at a melt temperature of 130 °C and rotation speed of 400 rpm. The screw diameter and the L/D ratio were 25 mm and 30, respectively. The extrudates were cooled in a water bath and pelletized. Injection molding was carried out in a Battenfeld BA-230E reciprocating screw injection molding machine (Wittman, Wien, Germany) to obtain tensile (ASTM D638, type IV, thickness 1.84 mm) specimens. The screw of the plasticization unit was a standard screw with a diameter of 18 mm, L/D ratio of 17.8, and a compression ratio of 4. The melt temperature was 130 °C and the mold temperature was 15 °C. The injection speed was 10.2 cm^3^·s^−1^. The specimens were left to condition for 24 h in a desiccator before analysis or testing. The samples used for the electrical conductivity measurements, which were 70 mm in diameter and 1.1 mm thick, were obtained by hot-pressing at 130 °C using a Collin P200E press (Collin, Maitenbeth, Germany).

X-ray diffraction patterns were recorded in an X’pert X-ray diffractometer (PANalytical, Almelo, the Netherlands) at 40 kV and 40 mA, using a Ni-filtered Cu-K_α_ radiation source. The transmission electron microscopy (TEM) samples were ultrathin-sectioned at 30–40 nm using a cryo-ultramicrotome. The micrographs were obtained in a Tecnai G2 20 Twin microscope (FEI, Hillsboro, OR, USA) at an accelerating voltage of 200 kV.

For the electrical conductivity measurements, the electrical resistivity of the compression-molded sheets was determined and converted to conductivity values. Volume resistances were measured using a digital Keithley 6487 picoammeter (Keithley Instruments, Cleveland, OH, USA).

Tensile testing was carried out by means of an Instron 5569 machine (Instron, Norwood, MA, USA) at a cross-head speed of 50 mm/min and at 23 ± 2 °C and 50 ± 5% relative humidity. A minimum of five tensile specimens were tested for each reported value.

The melting and crystallization behavior of the NCs was studied by DSC in a DSC-7 calorimeter (PerkinElmer, Waltham, MA, USA) calibrated with an Indium standard. The samples were first heated from 0 to 100 °C at a heating rate of 20 °C/min, then cooled at the same rate and, subsequently, reheated in the same conditions. The melting temperature (T_m_) and enthalpy (ΔH_m_) of the bTPU were determined from the second heating scans using the peak maximum and area, respectively. The crystallization temperature (T_c_) was determined from the cooling scans. Dynamic mechanical analysis was carried out in a DMA Q800 apparatus (TA Instruments, New Castle, PA, USA) that provided the plots of the tanδ against temperature. The scans were carried out from −100 to 60 °C at a constant heating rate of 4 °C/min and a frequency of 1 Hz.

## 3. Results and Discussion

### 3.1. Nanostructure

**Figure 1:**
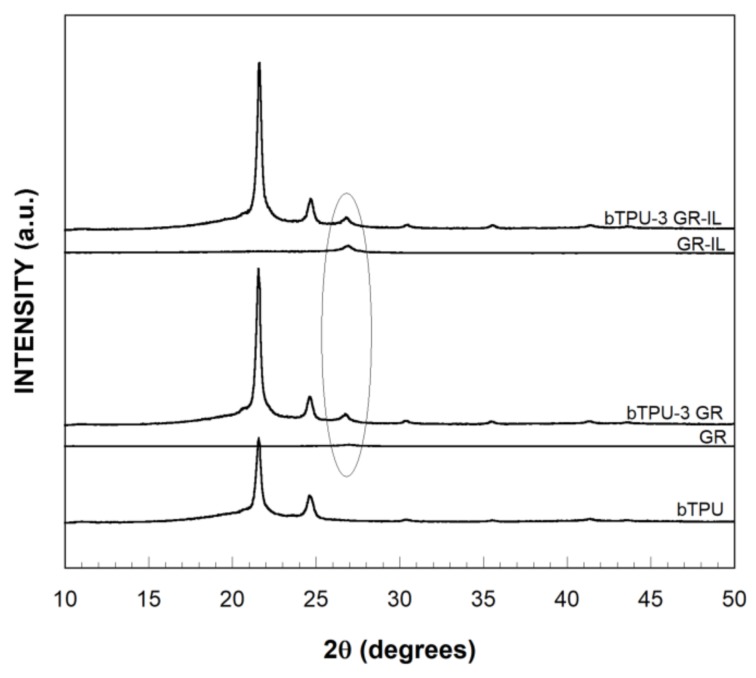
XRD diffractograms of the neat components and the bTPU-3 GR and bTPU-3 GR-IL NCs. The circle indicates the typical position of the corresponding peak of ordered graphene.

The nanostructure of the NCs was analyzed by XRD and TEM with the aim of ascertaining the effect of the modification of the IL on the dispersion level of the GR. Figure 1 shows the XRD diffractograms of the bTPU-3 GR and bTPU-3 GR-IL NCs, as well as those of the neat components. As the section of the figure marked with a circle shows, the diffractograms of the neat GR and GR-IL do not show the characteristic strong, narrow peak of graphite (2θ ≈ 26°, not shown in the figure), which corresponds to the reflection of the 002 planes of well-ordered sheets [1,39,40]; instead, a small peak of very low intensity appears in the same position. This indicates the absence of ordered structures, and the exfoliated nature of GR and GR-IL [26,41]. In the case of the NCs, the intensity of this peak is slightly higher, which could be indicative of the formation of some ordered structures or the stacking of GR sheets in the NCs during melt mixing [26], caused by the considerable van der Waals forces and strong π–π interactions between the graphene sheets [41,42,43,44]. However, the low intensity of these peaks points to a minor presence of ordered graphene sheets in the NCs.

**Figure 2:**
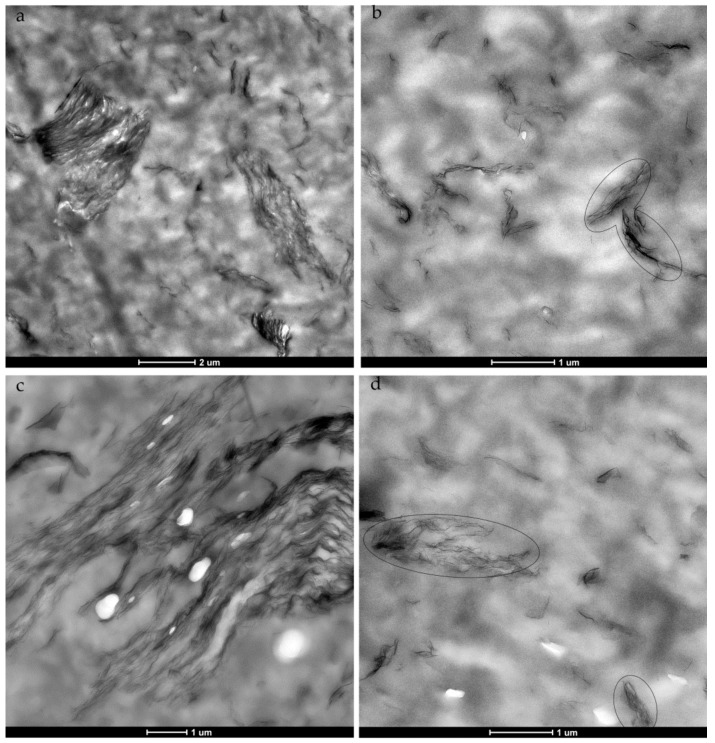
TEM micrographs of the bTPU-3 GR (**a**,**b**) and bTPU-3 GR-IL (**c**,**d**) NCs, obtained with low (left) and high (right) magnifications. Some graphene stacks have been highlighted with a circle in figures (b,d).

Additional information about the degree of dispersion of the nanofillers and the nanostructure of the NCs was obtained by TEM, and micrographs of the bTPU-3 GR and bTPU-3 GR-IL NCs are shown, as an example, in Figure 2. Other compositions showed similar nanostructures. As can be observed, the dispersion of both GR and GR-IL is nonuniform, presenting both relatively large stacks and stacks with only a few layers (Figure 2a,c), as well as individually dispersed graphene sheets (Figure 2b,d). The nanostructure of these bTPU NCs is similar to that observed for other NCs obtained by melt-mixing [26,33,45,46,47] and, taking into account that the samples for microscopy were prepared in perpendicular planes with respect to the flow direction, the GR platelets seem to have oriented in the flow direction during mold-filling [26,47,48]. Although complete exfoliation was not achieved, the dispersion of GR and GR-IL in the bTPU NCs was good enough to lead to significant improvements in the mechanical and electrical properties, as discussed below.

Regarding the effect of the IL-modified GR, the nonuniform dispersion makes it impossible to detect any significant difference in the degree of dispersion between the GR and the GR-IL. However, electrical conductivity is very sensitive to the dispersion level of nanofillers throughout the matrix and can be used as a qualitative measurement of this parameter.

**Figure 3:**
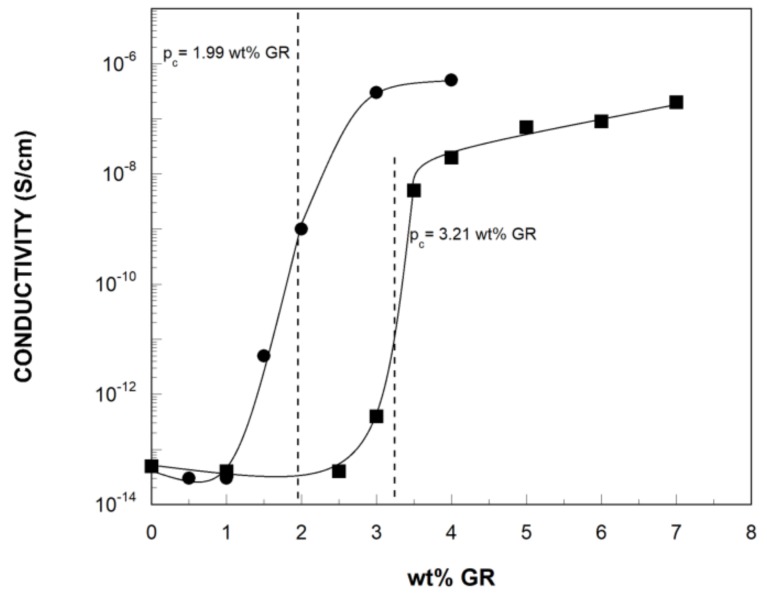
Electrical conductivity of the bTPU/GR (■) and bTPU/GR-IL (●) NCs as a function of the GR content. The dotted lines mark the calculated p_c_.

Figure 3 shows the electrical conductivity of the bTPU NCs as a function of the GR content. Up to a graphene concentration of 2.5 wt% in bTPU/GR and 1 wt% in bTPU/GR-IL NCs, the electrical conductivity remained almost unchanged, but when the graphene concentration exceeded these contents, the electrical conductivity increased dramatically: from 5 × 10^−14^ S/cm for the neat bTPU to 2 × 10^−7^ S/cm for the bTPU-7 GR NC and to 5 × 10^−7^ S/cm for the bTPU-4 GR-IL, i.e., 7 orders of magnitude.

The electrical percolation threshold (p_c_) was fitted using the power law function for conductivity values near the p_c_ [49]: σ(p) = B(p-p_c_)^t^, where σ(p) is the experimental conductivity value at concentrations p > p_c_, B is the proportionality constant, and t is the critical exponent. The experimental results were fitted and the p_c_ was determined at 3.21 wt% GR for the bTPU/GR NCs and at 1.99 wt% GR for the bTPU/GR-IL NCs. Therefore, modifying the GR with the IL leads to a significant reduction in the percolation threshold of the bTPU NCs. In addition, when conductive NCs with the same amount of GR are compared (for example, bTPU-4 GR and bTPU-4 GR-IL), the latter showed a significantly higher electrical conductivity. ILs are known to interact with the graphene by cation–π stacking and π–π interactions which prevent reaggregation of graphene sheets [9,10]. Their ability to improve the dispersion of graphene in several thermoplastic matrices has been demonstrated [7,9,11,12,13,14]. Therefore, it can be concluded that, although not clearly detected by TEM, the modification of GR with IL leads to the enhanced dispersion of the nanofiller, which, in turn, gives rise to a significantly lower electrical percolation threshold in the bTPU/GR-IL NCs than in the bTPU/GR NCs.

In spite of the important contribution of graphene as a conductive nanofiller, relatively few research studies have analyzed its effects on the electrical conductivity of this type of polymer NCs. When compared with other TPU NCs obtained by melt blending, the p_c_s obtained in this work are lower than those observed in a study on TPU NCs with expanded graphite [28]. In the same work, the p_c_ was reduced to 2 wt% upon optimization of the processing parameters; however, the NCs obtained presented an inhomogeneous nanostructure featuring large elongated aggregates, which is unsuitable from a mechanical point of view. Kim et al. [26] reported that the surface resistance of melt-processed TPU NCs with thermally-reduced graphite oxide (TRG) decreased at contents >0.5 vol% (~0.9 wt%). This finding is similar to the one obtained for the bTPU/GR-IL NCs in this work, where the conductivity increased at GR contents greater than 1 wt% (Figure 3). However, it should be mentioned that the value reported by Kim et al. refers to surface conductivity, and not volume conductivity as was measured in this work.

Higher [1,50,51,52,53] p_c_ values have been reported for melt-mixed NCs based on other polymer matrices and graphene. As in other NCs with carbonaceous nanofillers [54], the p_c_ is affected by various factors including the aspect ratio, the degree of dispersion or orientation of the nanofiller, the processing method and parameters, and the viscosity, molecular weight, and crystallinity of the polymer matrix.

In conclusion, the modification of GR with IL is an effective method of reducing considerably the electrical percolation threshold in polymer/graphene NCs, as the p_c_ determined in this study is one of the lowest electrical percolation thresholds reported in the literature for directly melt-blended NCs with a graphene-like nanofiller.

### 3.2. Mechanical Properties

**Figure 4:**
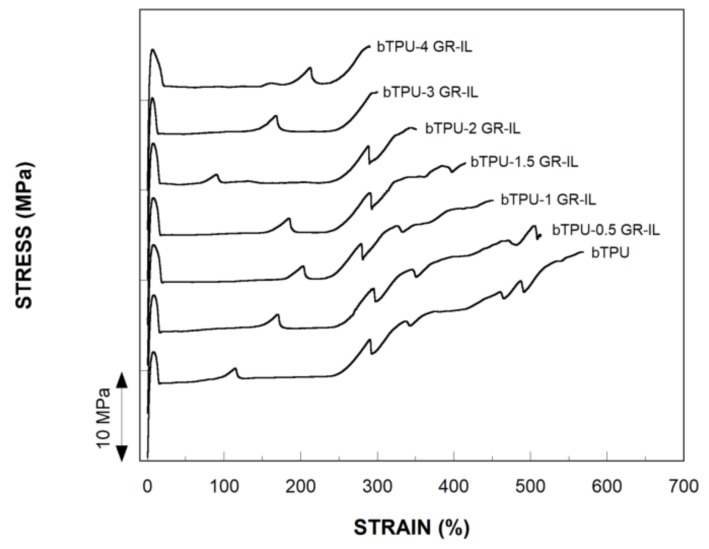
The stress–strain curves of the bTPU/GR-IL NCs. The curves are shifted along the vertical axis.

As TPUs are used for a wide range of different applications, optimum control of their mechanical properties is critical. However, the stiffness of TPUs is low, so improving Young’s modulus while retaining other desirable properties is an objective of particular interest. Therefore, the effects of the addition of GR and GR-IL on the mechanical properties of the bTPU were analyzed by tensile testing and the obtained results are summarized in Table 2. As the bTPU/GR NCs showed similar behavior, only the stress–strain curves of the neat bTPU and the bTPU/GR-IL NCs are shown in Figure 4. As can be observed, all the samples showed yielding and broke at high elongation values.

**Figure 5:**
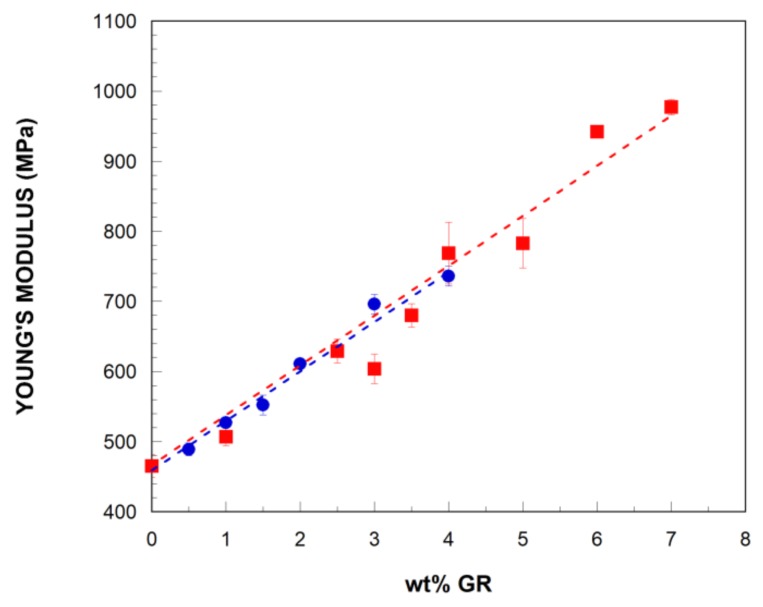
Young’s modulus of the bTPU/GR (■) and bTPU/GR-IL (●) NCs as a function of the GR content.

Figure 5 represents the Young modulus of the bTPU NCs as a function of the GR content. As can be seen, it increased almost linearly with the addition of GR and GR-IL. The linear modulus increase suggests that the dispersion and exfoliation levels of GR and GR-IL do not depend on their content in the NCs. As Figure 5 also reflects, very slight differences can be observed in the behavior of the GR and GR-IL NCs. This is a particularly positive result in the IL-modified NCs, because ionic liquids are known to act as plasticizers when added to polymeric matrices [55,56] and the IL used in this work did plasticize the bTPU matrix (see Table S1). Moreover, when the modulus increase per unit of added graphene was calculated, slightly higher values were obtained in the GR-IL NCs than in the GR NCs (average of 69 MPa vs. 65 MPa, respectively). Although the difference is in most cases close to the experimental error of measurement, it is deemed significant because the yield strength values showed a similar, even clearer trend, as discussed below. This enhanced mechanical behavior also suggests improved dispersion in the IL-modified GR, as already stated from the electrical conductivity measurements, which leads to greater reinforcing efficiency than the unmodified GR.

The reinforcing effect of graphene in melt-mixed TPU NCs has been previously analyzed in the literature [24,26,27,28,29]; the extent of the increase in Young’s modulus varies considerably in the studies. Maximum increases of 558% and 164% have been reported for TPU NCs with thermally-reduced graphite oxide [26] and expanded graphite [28], respectively. However, the modulus of the neat TPU in the referenced studies (6 and 27 MPa) was much lower than that of the bTPU used in this work (465 MPa), which would limit its applicability considerably. In addition, in the case of the TPU NCs with expanded graphite [28], the increase in Young’s modulus was not due only to the reinforcing effect of the expanded graphite, but also to the greater crystallinity of the TPU in the NCs.

According to the bibliography, several factors may affect the stiffening effect of graphene-based nanofillers in polymer matrices, including the dispersion level, aspect ratio, the concentration, interface bonding, etc., [44], which helps to explain the broad range of modulus increases found in the literature. In the studies where graphene was melt-mixed with a variety of polymer matrices [46,47], the polarity match between the polymer and the filler, the nature of the polymer, and the processing conditions have all been observed to affect the degree of dispersion of the graphene and, thus, the enhancement in properties [46,47].

**Figure 6:**
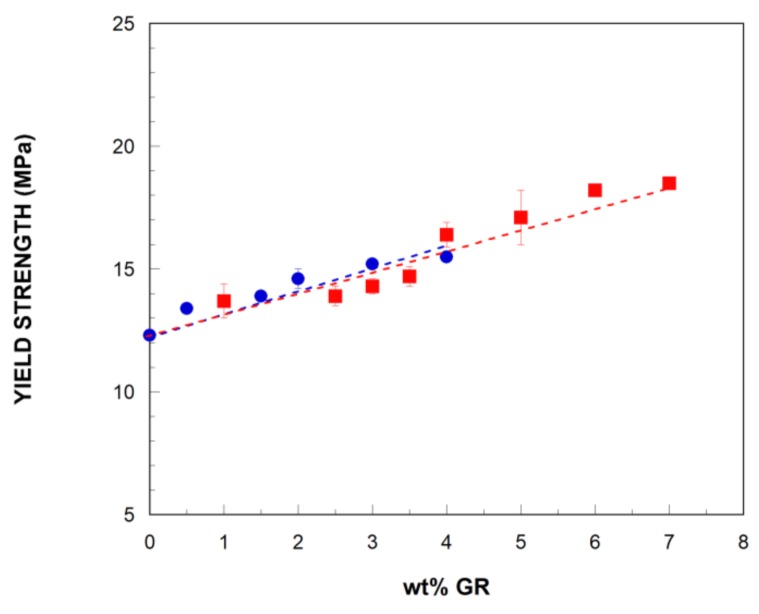
The yield strength of the bTPU/GR (■) and bTPU/GR-IL (●) NCs as a function of the GR content.

As can be observed in Figure 4, all the compositions broke during the strain hardening stage of the stress–strain curves, and the tensile strength value corresponds to the stress at break. However, the elongation at break of the NCs decreased, as discussed below, at increasing GR contents, which is reflected in the tensile strength values (Table 2). This has also been observed in other TPU/graphene NCs [28]. In order to analyze the effect of adding the GR, the yield strength of the bTPU NCs as a function of the GR content is presented in Figure 6, and the values are displayed in Table 2. Both NCs showed linear increases as the nanofiller content increased, but, as can be seen in the figure and was previously discussed, modifying GR with IL leads to enhanced yield strength compared with the unmodified GR. Young’s modulus and yield strength usually show similar behaviors, as they do in the present work, but the positive effect of the GR-IL is even clearer in the latter than in the former. In fact, the average yield strength increase per unit of added graphene is 37% higher in the GR-IL NCs than in the GR NCs (1.18 MPa vs. 0.86 MPa, respectively).

**Figure 7:**
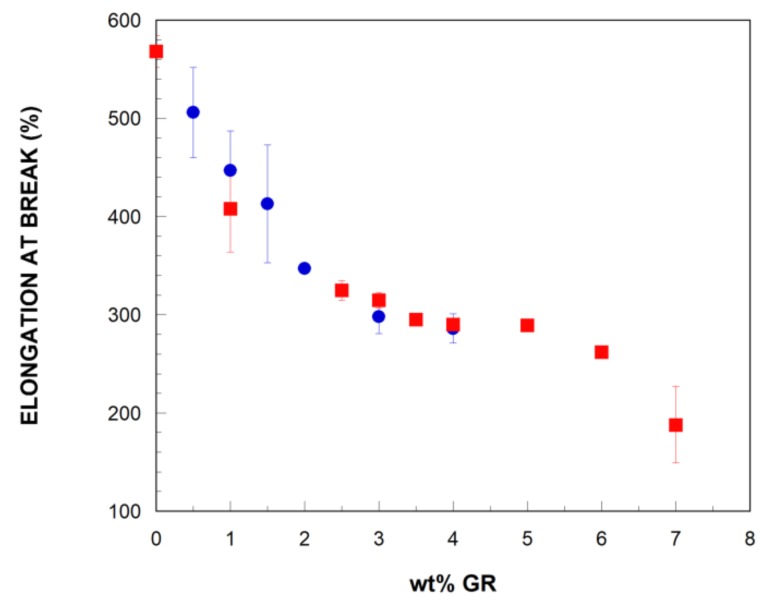
Elongation at break of the bTPU/GR (■) and bTPU/GR-IL (●) NCs as a function of the GR content.

Finally, and as mentioned in the previous paragraph, the ductility, measured as the elongation at break, of the bTPU (Figure 7) decreased significantly with a minimum addition (1 wt%) of GR, and more slowly at higher GR contents. Similar behavior has been reported for other melt-mixed TPU/graphene NCs [24,28,29]. However, in this work, all the NCs showed a clearly ductile nature, with elongation at break values above 200%; however, the high standard deviation of the measurements does not allow for any conclusions to be drawn regarding the effect of modifying GR with IL on ductility.

### 3.3. Thermal Properties

**Figure 8:**
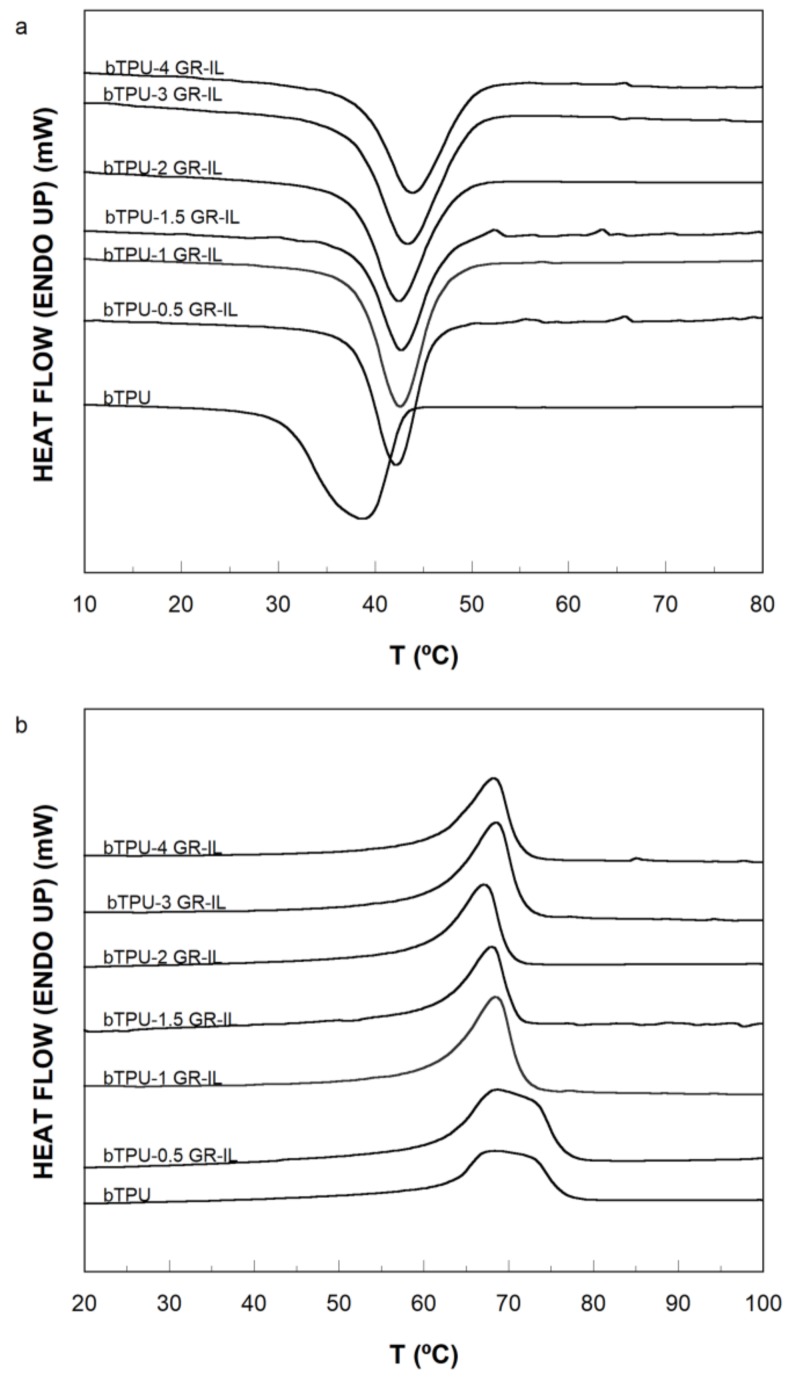
Cooling (**a**) and second heating (**b**) thermograms of the bTPU/GR-IL NCs.

Figure 8 shows the DSC cooling (a) and second heating (b) curves of the neat bTPU and the bTPU/GR-IL NCs. The curves corresponding to the bTPU/GR NCs are not presented as they show a similar trend. The crystallization temperatures (cooling scans), melting temperatures, and enthalpies (heating scans) of the bTPU NCs are shown in Table 3.

As can be seen in Figure 8a, a broad crystallization peak appeared on cooling at between 30 and 40 °C in the neat bTPU, indicating a broad distribution of the perfection/size of the crystals, which is attributed to the hard segments of the bTPU. In both the bTPU/GR and the bTPU/GR-IL NCs, the crystallization exotherms narrowed and shifted to higher temperatures (Table 3) as a result of the nucleating effect of the GR [1,51]. The increase was pronounced at low GR contents, but T_c_ remained unchanged as greater amounts of GR were added.

With respect to the second heating scans (Figure 8b), the neat bTPU showed a very broad melting endothermic peak at ~64–74 °C. The characteristics of this melting endotherm indicate a broad distribution of crystal sizes, fully consistent with the corresponding exotherm in Figure 8a, attributed to the crystallization of the hard segments of the bTPU matrix. The melting endotherms were narrower for the NCs and the main peaks appeared at the lowest values of the melting interval of the neat bTPU endotherm. This indicates a more homogeneous population of crystal sizes in the NCs [51] and a smaller mean size of the crystals. As previously proposed for the cooling curves, this is probably related to the nucleating effect of the GR on the crystallization of the bTPU.

With respect to the melting (Table 3) or crystallization (not shown) enthalpies, they hardly changed when the GR content changed. Different behaviors have been observed in the crystallinity of the polymer matrix of NCs when graphene is added: (i) increases in crystallinity resulting from a strong nucleation effect [1,40] (although the changes were not significant enough to affect the mechanical properties [57]); (ii) decreases in crystallinity as graphene acts as a physical barrier due to its large surface area and causes interference in the crystallization process [47,51,58]; and (iii) no change [59]. The behavior observed in Table 3 seems to indicate that even though GR favors the formation of bTPU crystal sites, the degree of crystallinity of the bTPU is unaffected by its presence.

The glass transition temperatures of the soft segment phase of the bTPU were determined from the tanδ curves obtained by DMTA and the results are summarized in Table 3. The T_g_ of unfilled bTPU was located at −16.0 °C and the incorporation of either GR or GR-IL gave rise to a scarcely significant increase of 1 to 1.5 °C, close to the experimental error of the measurement. While the presence of graphene-like structures has, on occasion, been reported to have hindered the molecular mobility of the polymer matrix causing an upward shift in T_g_ [58,60], in most studies the T_g_ remained unchanged upon the addition of graphene [1,40,51,61].

## 4. Conclusions

Electrically and mechanically reinforced biobased thermoplastic polyurethane (bTPU)/graphene nanocomposites (NCs) were successfully obtained using conventional melt-mixing equipment. The modification of GR with an IL using a simple and rapid method in order to improve the dispersion level of the nanofiller was successful since it reduced the electrical percolation threshold and enhanced the mechanical behavior.

NCs with either GR or GR-IL presented nonuniform dispersion, featuring both relatively large stacks and stacks with a low number of layers, as well as individually dispersed graphene sheets. The modification of GR with IL led to an improved dispersion level, as the percolation threshold of the bTPU/GR-IL NCs was significantly lower (1.99 wt%) than that of the bTPU/GR NCs (3.21 wt%). In addition, the NCs with GR-IL showed higher electrical conductivity values than the bTPU/GR NCs.

The addition of either GR or GR-IL led to significant improvements in Young’s modulus and yield strength. The superior mechanical behavior of the modified GR-IL NCs with respect to the unmodified GR NCs—which was only slightly noticeable in the case of Young’s modulus but more significant in the yield strength—lends further support to the improved level of dispersion of the nanofiller. Although the ductility values of neat bTPU could not be maintained in the NCs, all NCs showed elongation at break values of over 200%.

The NCs showed a more homogeneous population of crystal sizes and a smaller mean size of crystal than the unfilled bTPU, due to the nucleating effect of the GR. However, the degree of crystallinity of the bTPU in the NCs remained unchanged. The glass transition temperature of the bTPU was also unaffected by the addition of GR. No differences in thermal properties were observed between the GR and the GR-IL NCs.

## Figures and Tables

**Table 1:** Prepared biobased thermoplastic polyurethane (bTPU) polymer nanocomposites (NCs) and their total nanofiller and graphene (GR) contents.

Composition	Nanofiller wt%	GR wt%
bTPU-0.5 GR-IL	1	0.5
bTPU-1 GR-IL	2	1
bTPU-1.5 GR-IL	3	1.5
bTPU-2 GR-IL	4	2
bTPU-3 GR-IL	6	3
bTPU-4 GR-IL	8	4
bTPU-1 GR	1	1
bTPU-2.5 GR	2.5	2.5
bTPU-3 GR	3	3
bTPU-3.5 GR	3.5	3.5
bTPU-4 GR	4	4
bTPU-5 GR	5	5
bTPU-6 GR	6	6
bTPU-7 GR	7	7

**Table 2:** Mechanical properties of the bTPU NCs.

Composition	Young’s Modulus (MPa) (±10 MPa)	Elongation at Break (%) (±20%)	Tensile Strength (MPa) (±0.5 MPa)	Yield Strength (MPa) (±0.5 MPa)
bTPU	460	560	24.0	12.5
bTPU-0.5 GR-IL	490	500	20.5	13.5
bTPU-1 GR-IL	530	440	18.5	14.0
bTPU-1.5 GR-IL	550	420	18.0	14.0
bTPU-2 GR-IL	610	340	16.5	14.5
bTPU-3 GR-IL	700	300	15.0	15.0
bTPU-4 GR-IL	740	280	14.5	15.5
bTPU-1 GR	510	400	18.0	14.0
bTPU-2.5 GR	630	320	16.5	14.0
bTPU-3 GR	600	320	16.0	14.0
bTPU-3.5 GR	680	300	16.0	14.5
bTPU-4 GR	770	280	17.0	16.5
bTPU-5 GR	780	280	17.0	17.0
bTPU-6 GR	940	260	15.5	18.0
bTPU-7 GR	980	180	16.0	18.5

**Table 3:** The crystallization temperature (T_c_), melting temperature (T_m_), melting enthalpy (∆H_m_), and glass transition temperature (T_g_) of the bTPU NCs.

Composition	T_c_ (°C) ^a^ (±0.5 °C)	T_m_ (°C) ^a^ (±0.5 °C)	ΔH_m_ (J/g) ^a^ (±5 J/g)	T_g_ (°C) ^b^ (±0.5 °C)
bTPU	38.5	64.0–74.0	70	−16.0
bTPU-0.5 GR-IL	42.0	68.5	70	−16.0
bTPU-1 GR-IL	42.5	69.0	70	−15.5
bTPU-1.5 GR-IL	42.5	68.0	70	−15.5
bTPU-2 GR-IL	42.5	67.0	70	−15.5
bTPU-3 GR-IL	43.5	68.0	70	−15.0
bTPU-4 GR-IL	44.0	68.5	70	−15.0
bTPU-1 GR	42.0	66.5	75	−15.0
bTPU-2.5 GR	40.5	66.0	75	−15.0
bTPU-3 GR	44.5	66.5	70	−15.0
bTPU-3.5 GR	43.5	67.5	70	−14.5
bTPU-4 GR	44.0	67.5	75	−15.0
bTPU-5 GR	43.5	68.0	75	−16.0
bTPU-6 GR	44.0	66.0	75	−15.0
bTPU-7 GR	44.0	68.0	75	−14.5

^a^ obtained by DSC. ^b^ obtained by DMTA.

## Supplementary Materials

The following are available online at https://www.mdpi.com/2073-4360/11/3/435/s1, Table S1: Young’s modulus of the bTPU/IL blends.
