# Supplementary material for: Using an Ionic Liquid to Reduce the Electrical Percolation Threshold in Biobased Thermoplastic Polyurethane/Graphene Nanocomposites
Source: Polymers (Basel). 2019 Mar 6;11(3):435. doi: 10.3390/polym11030435 (PMC6473422; doi:10.3390/polym11030435)
Supplement: Supplementary file 1 [file polymers-11-00435-s001.pdf]

Table S1. Young's modulus of the bTPU/IL blends.

COMPOSITION	YOUNG'S MODULUS (MPa) (±10 MPa)
bTPU	460
bTPU-1 IL	460
bTPU-2 IL	450
bTPU-3 IL	440
